# Role of metallothioneins in benign and malignant thyroid lesions

**DOI:** 10.1186/1756-6614-5-26

**Published:** 2012-12-28

**Authors:** Bartosz Pula, Pawel Domoslawski, Marzena Podhorska-Okolow, Piotr Dziegiel

**Affiliations:** 1 Department of Histology and Embryology, Medical University in Wroclaw, Wroclaw, Poland; 2 Department of General, Gastroenterological and Endocrinological Surgery, Medical University in Wroclaw, Wroclaw, Poland; 3 Department of Physiotherapy, Wroclaw University School of Physical Education in Wroclaw, Wroclaw, Poland

**Keywords:** Metallothionein, Thyroid, Nodular goiter, Cancer

## Abstract

Recent findings in the past two decades have brought many insights into the biology of thyroid benign and malignant lesions, in particular the papillary and follicular thyroid cancers. Although, much progress have been made, thyroid cancers still pose diagnostic problems regarding differentiation of follicular lesions in relation to their aggressiveness and the treatment of advanced and undifferentiated thyroid cancers. Metallothioneins (MTs) were shown to induce cancer cells proliferation, mediate resistance to apoptosis, certain chemotherapeutics and radiotherapy. Therefore, MTs may be of utility in diagnosis and management of patients with benign and malignant lesions of the thyroid.

## Introduction

Thyroid cancer is regarded as the most common cancer of the endocrine organs and among all cancers it accounts approximately up to 2.5% of all malignancies [1,2]. Although, the death rates remain relatively low, there is a growing trend towards higher incidence of thyroid cancer which might not be explained by progress in detection of even relatively small thyroid cancers [1,3,4]. The majority of cancers of this organ develop from follicular epithelial cells and due to their morphology and biology are divided grossly into two groups. The first group, the well differentiated thyroid cancers, which include the papillary thyroid carcinoma (PTC) and the follicular thyroid carcinoma (FTC) are usually slow growing, characterized by iodine intake and susceptible to TSH-suppressive therapy [5-7]. The PTC accounts for approximately up to 80% of thyroid tumours, whereas the FTC accounts for 5-15% of diagnosed thyroid malignancies [8]. These cancers are usually curable utilizing combined surgical and radioiodine therapy in contrast to the anaplastic thyroid cancer (ATC), which represents the poorly differentiated thyroid cancers [7]. ATC comprises only 1-2% of all diagnosed thyroid malignancies, but is characterized by rapid and invasive growth resulting in fulminant disease course and poor outcome [7,9]. In thyroid, approximately 5% of thyroid malignancies are diagnosed as medullary carcinoma, which in turn originates from the parafollicular C cells [7].

All the above mentioned histological variants of thyroid carcinomas may be easily distinguished upon histopathological examination, however in case of FTC problems may occur in distinguishing these tumors from benign follicular adenomas (FA) and follicular variants of PTC (FVPTC) [6]. In case of FTC and FA the differential diagnosis is made by identification of signs of invasiveness into the tumour capsule in numerous serial hematoxyline and eosine stained slides. This renders the fine-needle aspirates (FNA) of thyroid follicular nodules useless for diagnostic purposes and biopsies not allowing for determination of the lesions malignant potential are determined as of indeterminate significance [5]. Up to 40% of FNA are regarded as indeterminate and subsequently the majority of the patients undergo surgery, but only the minority of the resected thyroid nodules are classified as malignant [10-14]. Moreover, no immunohistochemical markers of invasiveness in FNA have been so far discovered [5,15]. Molecular testing of FNA and resected thyroid specimens may be of value, but none of the recently discovered mutations in thyroid tissue is widely used in the clinical practice [7,16].

Recent progress in molecular methods resulted in better understanding of the biology of thyroid tumours. It seems that metallothioneins (MTs), due to their characteristic properties may help in the differential diagnosis of thyroid tumours and patients management. This review presents the current knowledge regarding their expression in benign, as well as malignant thyroid lesions.

### Metallothionein structure, synthesis and biological functions

MTs are low molecular weight proteins (6-7kDa), which are expressed in almost all types of organisms [17-19]. MTs were first isolated in 1957 from horse renal cortex and since then our knowledge concerning these highly conserved proteins have evolved [18,20]. All MTs posses a highly conserved amino acid sequence and MTs isolated from different animal species show little structural changes. A single MT molecule consists of 61-68 amino acids, depending on the isoform, where up to one third of proteins sequence is composed of cysteine (Cys) residues [17,21]. Interestingly, this residues are organized in typical tandem sequences, with up to two other amino acids occurring between two cysteins [21]. Due to such high cysteine content, MT possess the ability to bind up to seven bivalent metal ions, such as zinc, copper, mercury, lead [19], Moreover, MTs may bind up to twelve univalent metal ions [19,22].

In the overall structure of MTs two domains have been recognized, the domain α and β [23]. The domain α comprises amino acids 31-68 and is located on the C-terminal edge, whereas the N-terminal domain β comprises amino acids 1-30 [20]. The latter due to its highest antigencity is used for production of antibodies, but due to high homology of particular MT isoforms, the utility of such obtained antibodies in distinguishing particular MT isoforms is therefore highly limited [20,24,25]. Although, MTs form a homogenous group of proteins (according to their amino acid sequence and overall structure leading to charge differences), four principal isoforms could be distinguished: MT-1, MT-2, MT-3 and MT-4 [26]. Most of our knowledge concerning MTs biology stems from research directed on examination of the MT-1 and MT-2 isoforms (MT-1/2), which are ubiquitously expressed in almost all cells of the organism [27]. Expression of MT-3 at first seemed to be more specific and restricted only to neurons and was first isolated from rat brain extracts suffering to experimental Alzheimer disease [28-30]. MT-3 is also known under the synonym growth inhibitory factor (GIF) as it experimentally blocked the regeneration of neurons in injured nervous tissue [30]. Recent studies have brought more insights into the biology of this protein, as it was also found to be expressed in some normal tissues as well as in different tumour types [31-37]. The expression of MT-4 seems to be restricted only to squamous epithelium of the skin and upper parts of the alimentary tract [38].

MTs are encoded by 17 genes located within the 16q13 region, from which 13 code for MT-1, two for MT-2 and one gene each coding for MT-3 and MT-4 [26,38,39]. However, at least 10 genes code for functional MT proteins: MT-1A, MT-1B, MT-1E, MT-1F, MT-1G, MT-1H, MT-1X, MT-2A, MT-3 and MT-4 [24,26]. Beside the functional isoforms, there were also seven non-functional isoforms identified: MT-1C, MT-1D, MT-1I, MT-1J, MT-1K, MT-1L and MT-2B [26,40]. MTs were found to be localized in cells cytoplasm, as well as in cell nucleus [20,41]. Isoforms of the MT-1 and MT-2 family were shown to be induced by several substances and agents e.g. heavy metals, steroids, cytokines, growth factors and free radicals [42-46]. Zinc ion seems to represent the natural biological compound responsible for the induction of MTs expression. Zinc ions bind to the metal response element-binding transcription factor (MTF-1), which interacts *via* its zinc finger domains with a particular DNA region - the so called metal response element (MRE). Binding of MTF-1 to MRE results in initiation of MT-1/2 gene transcription [47-49]. Metal ions, other than zinc, induce MT-1/2 expression independently of the binding to the MTF-1 transcription factor as they may not activate this protein. However, these metal ions possess higher affinity to MT-1/2 proteins as compared to zinc ions, what results with the release of the latter to the cytoplasm. As result, free zinc ions bind to the MTF-1, what activates the transcription of MT-1/2 genes [50-52]. Similar mechanism is observed during oxidative stress, in which MTs oxidation by hydrogen peroxide (H_2_0_2_) leads to release of zinc ions from these proteins [53,54]. Regulation of MTs expression by glucocorticoid hormones is regulated independently of the above mentioned mechanism, as glucocorticoid receptors bind to the specific regulatory sequence (GRE; glucocorticoid response element) in the promoter region of MTs genes activating their transcription [27,55]. MTs expression may be also induced during stress conditions *via* the antioxidant response element (ARE) [17,56,57].

Although, MT-3 shows approximately 70% sequence similarity to other MTs, however there are some differences in its structure, what might reflect its functional diversity in comparison to other MTs. MT-3 posses a glutamate-rich hexapeptide near the C-terminus and contains a CPCP (Cys-Pro-Cys-Pro) motif (amino acids 6-9), which is absent in other MTs [29,58]. This might be reflected by the unique growth inhibitory role of MT-3, not apparent in other MTs [28-30,58].

### Metallothioneins in neoplasia

Lines of evidence point to significant role of MTs of the MT-1 and MT-2 isoforms in development and progression of numerous neoplastic diseases [18,41,59,60]. Due to the metal binding properties of MTs, these proteins may act as possible zinc ions donors for zinc-dependent enzymes and transcription factors, which play crucial role in processes such as replication, transcription and translation [18,61]. It was shown, that in inactive cells (G0 phase) MTs can be detected in the cytoplasm, whereas in cells undergoing division MTs are shifted to the nucleus. In addition, the high cytoplasmic expression of MTs is noted at the end of G1 phase and at the G1/S threshold, while the highest MTs concentration in cell nucleus was noted in the S and G2 phases [62,63]. The translocation of MTs into the nucleus during G1/S phase in tumour cells suggests that MTs facilitates cell proliferation by donating zinc ions to various transcription factors. Moreover, numerous reports based on immunohistochemical methods seemed to confirm the results obtained *in vitro* suggesting the role o MTs in cell cycle regulation, as MTs expression was detected in the cell cytoplasm and nucleus in organs undergoing growth or regeneration (e.g. liver, kidney and parabasal cells of stratified epithelium) [64-67]. In addition, MTs overexpression is frequently observed in various malignancies (epithelial as well as mesenchymal tumours) and in some cases increased with growing malignancy grade of those tumours [20,24,66-75]. Lines of evidence suggest, that MTs may diminish the suppressor function of the p53 protein leading to uncontrolled growth and proliferation [61,76]. Numerous experimental data have, shown a positive correlation of MT expression and Ki-67 or PCNA (proliferating cell nuclear antigen) antigens in human tumour tissues, supporting the pro-proliferative role of MTs [24,25,59,68,69,77,78].

Higher expression of MTs in human tumours was also linked to increased chemoresistance of some tumour types (e.g. gastric, ovarian, breast cancer), as it levels increased after chemotherapy with agents such as cis-platin, bleomycin, irinotecan or cyclophosphamide [41,79-81]. Due to the structure of MTs, they may diminish the effects of agents inducing the oxidative stress (daunorubicin, doxorubicin), in mechanism involving inactivation of free radicals [20,82]. Thanks to the high affinity of MTs to metal ions, these proteins may also inactivate alkylating drugs, characterized by cytotoxic effects dependent on heavy metal compounds (e.g. cis-platin, carboplatin) [83]. Several studies have shown that MTs may mediate cancer cells chemoresistance in human malignant tumours characterized by MTs overexpression [79,81,84-86]. Moreover, free radicals scavenging properties MTs may also take part in cancer cells resistance to radiotherapy [87].

Most of the studies have revealed, that MT-1/2 overexpression in malignant cells is associated with poor survival of patients with e.g. non-small cell lung cancer, intrahepatic cholangiocarcionoma, synovial sarcoma, renal cancer or melanoma [24,70,71,88-90]. However, some of the studies have showed that MTs in some tumour types do not yield any prognostic significance, or - in some case - were even associated with patients better prognosis [72,91]. Interestingly, in some studies MT-1G (hepatocellular and papillary thyroid cancer), MT-1F (colorectal cancer) and MT-3 (gastric and esophageal cancer) isoform were regarded as potent tumour suppressors [92-97]. In spite of many efforts, the role of MT-3 in neoplastic disease remains ambiguous and the results of the studies were frequently inconsistent [31,32,36,37,98,99]. Therefore it seems, that the role of particular metallothionein isoforms differ significantly and the utilization of MTs as potent prognostic factors in human cancers requires further research.

### Metallothionein expression in thyroid tissues

**Figure 1:**
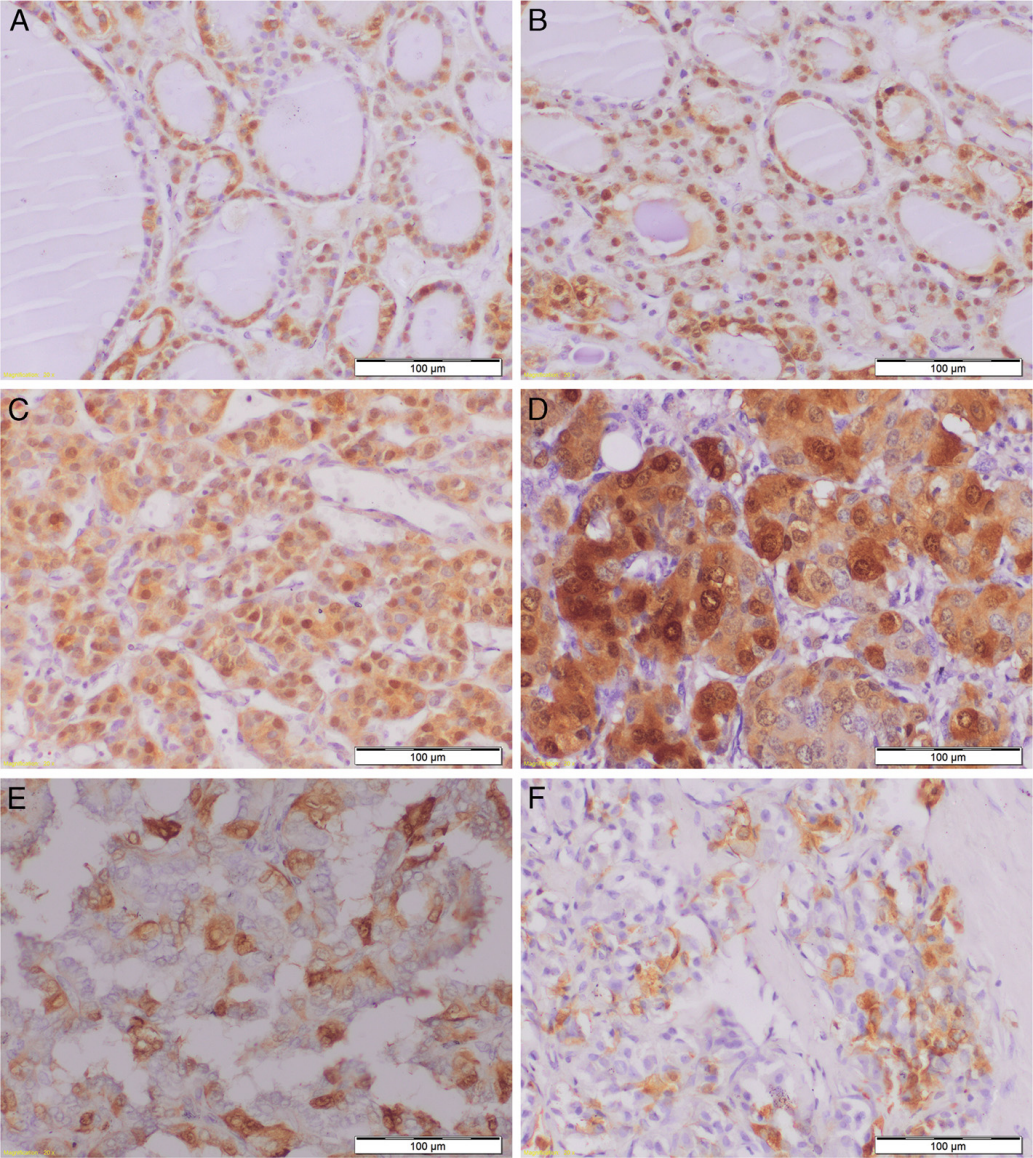
**Differentiated nyclear cytoplasmic MT-1/2 expression in normal thyroid cells (A), nodular goiter (B), follicular adenoma (C), follicular carcinoma (D), papillary carcinoma (E) and medullary carcinoma (F). **Magnification ×200.

First study regarding MTs expression in human thyroid tissue was performed by Nartney *et al.* in 1987, who used a polyclonal rabbit anti-MT antibody to examine the expression levels of MTs in surgically resected tumour samples and normal thyroid tissue biopsied from autopsy cases [100]. This pilot study revealed that in paraffin embedded tissues of normal thyroid, only 20% of analyzed cases expressed MTs in the nucleus of follicular thyroid cells, whereas a nuclear-cytoplasmic expression pattern of MT was noted in majority of the surgically resected tumour samples (91%) [100]. The results obtained by Nartney *et al.* regarding MTs expression in normal thyroid tissues were not in accordance with the results obtained by other studies, which showed that MTs expression is mostly downregulated in thyroid cancers when compared to normal thyroid follicular cells or benign lesions of the thyroid e.g. nodular goiter (Figure 1) [15,92,101,102]. These studies were performed either on paraffin-embedded tissues using immunohistochemistry or on fresh frozen material utilizing expression microarrays or real-time PCR. The latter allowed to distinguish the expression of particular functional MTs isoforms - data not possible to obtain with currently available antibodies due to the homogenous structure of MTs isoforms [92,101,102]. 

The microarray studies identified MT-1G gene expression as downregulated in PTC and FTC, supporting recent reports of MT-1G suppressor role in human colon and hepatocellular carcinoma [91-93,101,102]. Lines of evidence suggest that hypermethylation of gene promoters occurs frequently in thyroid cancers and may be responsible for altered expression of sodium-iodide symporter (NIS), proteins associated with adhesion or cell cycle [103-105]. Moreover, treatment of thyroid cancer cell lines with demethylating agents could restore the dedifferentiation of thyroid cancer cell lines and their ability to iodide uptake [105]. The decreased expression of MT-1G in PTC was caused by the hypermethylation of the CpG islands, but not the loss of heterozygosity (LOH) [103]. This observations were also confirmed on cell lines derived from human PTCs: NPA-87 (poorly differentiated), K1 and K2 (well-differentiated) [103,106,107]. The treatment of K2 cell line, which showed the highest level of methylation, with demethylating agent 5-aza-2’-deoxycytidene and/or trichostatin A (a histone deacetylase inhibitor) induced the higher expression level of MT-1G [103]. Similar results were described with the K1 cell line [92].

Beside the MT-1G, also MT-1H and MT-1X were shown to be downregulated in human PTC and FTC [92]. Also in the study of Finn *et al.*, it was demonstrated that in PTCs with classic morphology, as well as in the FVPTCs, MTs expression was downregulated what points to a potential application of MTs as markers of thyroid malignancy [101]. Nevertheless, also in this aspect the biological significance of the above mentioned findings requires further research.

### Biological significance of MTs expression in thyroid cancers

The biological importance of MTs expression was only assessed in relation to the MT-1G isoform in the study of Ferrario *et al.*, in which the expression of this isoform was restored in the K1 thyroid papillary cancer cell line with use of a MT-1G-myc expression plasmid [92]. MT-1G introduction into the K1 cell line resulted in reduced growth rate in respect to control cells subsequently leading to formation of smaller colonies. The suppressory role of MT-1G was additionally confirmed *in vivo* in athymic nude mice, as K1 cells with MT-1G expression yielded reduced tumour growth as compared to control cells [92]. Downregulation of MTs in PTC may be also partly responsible for decreased inactivation of reactive oxygen species observed in this tumour type, what might contribute to the carcinogenesis process [108,109].

Impact of MTs expression on carcinogenesis of thyroid cancer was also analysed in both studies of Liu *et al*. with regard to papillary (KAT5 cells) and anaplastic thyroid cancer (ARO cell line) [110,111]. In both studies the cell lines were subjected to treatment with cadmium ions (a potent inductor of MTs expression), in order to examine the impact of MTs isoforms on carcinogenesis. Cadmium treatment led to the induction of MT-1 and MT-2 isoforms in both cell lines, although differences in MT profile were observed [110,111]. In the KAT5 cells, the MT-1G and MT-2A levels were identified as being the most abundant isoforms upon cadmium treatment, whereas in the ARO cell line additionally the MT-1A, MT-1F, MT-1H, MT-1X were identified as the main functional isoforms [110,111]. Both studies have shown that induction of those isoforms was accompanied by alteration in the cell cycle leading to increase of proportion of cells in the S and G2-M phase and decrease in the proportion of cells in the G0/G1 phase [110,111]. Moreover, cadmium treatment resulted also in rise of calcium ions influx and led to a moderate rise in the phoshorylated ERK1/2 (extracellular signal-regulated kinase), both processes capable of stimulating cells proliferation [112]. Although, it was shown that the expression of MT-1G may alter proliferative response of KAT5 cells, the results of both studies should be taken with caution, because the study design did not allow clear assessment of MTs role in the observed cell cycle alterations [92,110,111]. Moreover, in our own study we did not observe any significant correlation between the expression of the MT-1/2 and the proliferation antigen Ki-67 in the series of PTC and FTC cases [15].

### Clinical significance of MTs in thyroid

Although, the prognostic significance of MTs expression in thyroid cancer remains to be clarified, some of the obtained results may have potential to be to applied in clinical practice. Recent advance in molecular testing of thyroid carcinomas led to the identification of genes altered in particular type of the thyroid cancer [3,5,9,16]. It has become apparent that PTC and FTC harbour different mutations. PTC is characterized by the mutations in the RAS and BRAF genes as well as by rearrangements of the RET/PTC genes capable of activating the mitogen activated kinase (MAPK) pathway. In FTC alterations in the RAS gene and PAX8/PPARγ rearrangements were identified in majority of the cases [16]. However, these mutations were also shown to occur in benign thyroid lesions. This fact, on one hand confirms the multistep carcinogenesis process in the thyroid, but on the other may render difficulties in introducing these new findings into clinical practice [7]. MTs expression shown as downregulated in majority of the thyroid cancer studies, could be used as an ancillary marker helping in differential diagnosis of indeterminate FNA or surgical specimens of thyroid lesions [15,92,101-103]. We have recently shown that immunohistochemical examination of MT-1/2 expression may be of use in differentiating FA from FTC, which are impossible to distinguish in FNA examination [6,8]. The higher expression of MT-1/2 observed in FTC as compared to FA may be of clinical importance, although this finding must be confirmed on a larger patients cohort with establishing proper cut-off points for the diagnosis [15]. Moreover, the pronounced expression of MT-1/2 in FTC may be linked to resistance to radiotherapy.

## Conclusions

MTs due to their wide functional properties, potentially associated with progression of majority of human cancers may pose an interesting point of research in relation to human malignant thyroid lesions.

## Competing interests

The authors declare that no competing financial interests exist.

## Authors’ contributions

All the authors drafted the manuscript. All the authors read and approved the final manuscript.
